# Comparative Sensitivity of Rapid Antigen Tests for the Delta Variant (B.1.617.2) of SARS-CoV-2

**DOI:** 10.3390/v13112183

**Published:** 2021-10-29

**Authors:** Yuko Sakai-Tagawa, Seiya Yamayoshi, Peter J. Halfmann, Yoshihiro Kawaoka

**Affiliations:** 1 Division of Virology, Department of Microbiology and Immunology, Institute of Medical Science, University of Tokyo, Tokyo 108-8639, Japan; ytsakai@g.ecc.u-tokyo.ac.jp; 2 International Research Center for Infectious Diseases, Department of Special Pathogens, Institute of Medical Science, University of Tokyo, Tokyo 108-8639, Japan; 3 The Research Center for Global Viral Diseases, National Center for Global Health and Medicine Research Institute, Tokyo 162-8655, Japan; 4 Department of Pathobiological Sciences, School of Veterinary Medicine, University of Wisconsin-Madison, Madison, WI 53706, USA; peter.halfmann@wisc.edu

**Keywords:** SARS-CoV-2, COVID-19, rapid antigen test, sensitivity, delta variant, B.1.617.2

## Abstract

Rapid antigen tests (RATs) for COVID-19 based on lateral flow immunoassays are useful for rapid diagnosis in a variety of settings. Although many kinds of RATs are available, their respective sensitivity has not been compared. Here, we examined the sensitivity of 27 RATs available in Japan for the detection of the SARS-CoV-2 delta variant. All of the RATs tested detected the delta variant albeit with different sensitivities. Nine RATs (ESPLINE SARS-CoV-2, ALSONIC COVID-19 Ag, COVID-19 and Influenza A+B Antigen Combo Rapid Test, ImmunoArrow SARS-CoV-2, Fuji Dri-chem immuno AG cartridge COVID-19 Ag, 2019-nCoV Ag rapid detection kit, Saliva SARS-CoV-2(2019-nCoV) Antigen Test Kit, and Rabliss SARS-CoV-2 antigen detection kit COVID19 AG) showed superior sensitivity to the isolated delta variant. Although actual clinical specimens were not examined, the detection level of most of the RATs was 7500 pfu, indicating that individuals whose test samples contained less virus than that would be considered negative. Therefore, it is important to bear in mind that RATs may miss individuals shedding low levels of infectious virus.

## 1. Introduction

Severe acute respiratory syndrome coronavirus 2 (SARS-CoV-2) causes coronavirus disease 2019 (COVID-19). The WHO reported that more than 230 million cases of COVID-19, including approximately 4.8 million deaths, have occurred as of 29 September 2021 (https://covid19.who.int/). To reduce the burden by SARS-CoV-2, nonpharmaceutical interventions, vaccination, and patient treatment are required. For mitigation of infectious diseases, early and accurate patient diagnosis is essential.

For COVID-19 diagnosis, reverse transcription-quantitative PCR (RT-qPCR) using upper respiratory swabs or saliva has become the gold standard [1] because it possesses high sensitivity and specificity against the target agent. RT-qPCR is usually not available in local clinics where patients who suspect they have COVID-19 go first. Therefore, the collected specimens are transported to sites with RT-qPCR capability, resulting in delayed test results. To obtain results at local clinics, rapid antigen tests (RATs) for COVID-19 have become popular because RATs require just 15–30 min to give results. RATs are also helpful as screening tests for asymptomatic individuals since model analyses showed that population screening tests should prioritize frequency and turnaround time over sensitivity [2,3]. Therefore, RATs might be useful to reduce COVID-19 clusters and spread if frequent self-testing using RATs was performed before mass gatherings, domestic travel, or dining at restaurants. Although the sensitivity of RATs is lower than that of RT-qPCR [4,5,6,7,8,9,10,11], it is essential to utilize RATs with superior sensitivity for better detection. To achieve this aim, we [11] and other groups [12,13,14,15,16] compared the sensitivity of RATs using clinical specimens collected from COVID-19 patients who were infected with SARS-CoV-2 possessing aspartic acid or glycine at position 614 of the S protein (S-614D or S-614G). Recently, Jungnick et al. compared the sensitivity of four RATs for the alpha, beta, gamma, and delta variants [17]. Here, we examined the sensitivity of RATs available in Japan in September 2021 for the detection of the delta variant (lineage B.1.617.2) of SARS-CoV-2.

## 2. Materials and Methods

### 2.1. Biosafety Statements

All experiments with SARS-CoV-2 were performed in biosafety level 3 (BSL3) laboratories at the University of Tokyo, which were approved for such use by the Ministry of Health, Labour and Welfare, Japan.

### 2.2. Cells and Virus

Vero E6 cells expressing human serine protease TMPRSS2 (VeroE6-TMPRSS2) [18] were maintained in DMEM containing 10% fetal calf serum (FCS), 1 mg/mL G418, 100 units/mL penicillin, 100 µg/mL streptomycin, and 5 μg/mL Plasmocin prophylactic (InvivoGen, San Diego, CA, USA) and incubated at 37 °C under 5% CO_2_. SARS-CoV-2 (hCoV-19/USA/WI-UW-5250/2021, delta variant (lineage B.1.617.2) was propagated and titrated in VeroE6-TMPRSS2 cells.

### 2.3. RT-qPCR

Viral RNA was isolated from the specimens by using the QIAamp Viral RNA Mini Kit (QIAGEN, Tokyo, Japan). One step RT-qPCR was performed using the LightCycler 96 System (Roche Diagnostics, Tokyo, Japan) according to the protocol described earlier by the National Institute of Infectious Disease, Japan [19]. A Cq value of <40 was considered a positive result.

### 2.4. Rapid Antigen Test (RAT)

The RATs listed in Table 1 were evaluated according to the procedures described in the manufacturers’ instructions, using 75–75,000 plaque-forming units (PFU) of stock virus in a 50 μL volume. Two independent experiments were performed with each dilution.

## 3. Results

### 3.1. Comparison of Rapid Antigen Tests (RATs)

We evaluated 27 RATs that were available in Japan in September 2021 (Table 1). Of these 27 RATs (#1–17), 17 are approved for clinical diagnosis in Japan, whereas the other 10 RATs (#18–27) are not approved for such purpose in Japan. The 27 RATs are divided into three formats: the test strip format, the pen format, and the well format. In the test strip format, a test strip is soaked in lysis buffer containing the specimen or is dipped in the specimen and then soaked in the lysis buffer; the reaction occurs on the strip. In the pen format, the test strip is dipped into the specimen and the reaction occurs on the strip. This format allows saliva specimens to be loaded by holding the cartridge directly in the mouth. For the well format, lysis buffer containing the specimen is dropped into the well, and the reaction occurs inside a covered plastic body. The well format can be further subdivided into two groups based on how the result is evaluated; for tests #15, #16, and #17, a specific analyzer is required to evaluate the results, whereas the other well-format RATs are assessed by the human eye. Most RATs can process upper respiratory swabs including nasopharyngeal (NP), pharyngeal (P), oropharyngeal (O), or nasal (N) swabs, whereas saliva is the recommended sample for seven RATs (#19, #22, #23, #24, #25, #26, and #27) (Table 1). Tests #18, #20, and #21 can be used for both upper respiratory swabs and saliva. Since it is easy for individuals to collect nasal swabs and saliva, the RATs available for such specimens are suitable for self-testing.

All of the RATs we tested are immunochromatographic tests, meaning that their sensitivity is dependent on the binding kinetics and epitopes of the monoclonal antibodies used in each RAT, the composition of the lysis buffer, the volume of specimen used for analysis, and the method to visualize the result. We cannot directly compare the performance of monoclonal antibodies because the manufacturers do not disclose the properties or amino acid sequence of monoclonal antibodies; however, most RATs likely use monoclonal antibodies against the nucleoprotein of SARS-CoV-2. Because the amino acid sequences of nucleoprotein are similar among human betacoronaviruses, especially the subgenera sarbecovirus, cross-detection is likely to occur against SARS-CoV or SARS-CoV-2-related viruses such as RaTG13 and bat SARS-like coronaviruses. Most of the RATs claim cross-detection of SARS-CoV, with three exceptions: the manufacturers of tests #11 and #15 state that their tests show no cross-reactivity against SARS-CoV, and test #6 cross-detects a high concentration of human coronavirus HKU1 as well as SARS-CoV. Therefore, RATs that show cross-reactivity against SARS-CoV are not able to differentiate patients infected with SARS-CoV-2 and other sarbecoviruses under conditions where these viruses are co-circulating.

The amount of specimen used for each test varied between the RATs (Table 2). The input ratio for three RATs with the pen and test strip formats (#14, #22, and #26) was 100% because of the mechanism. Among the well-format tests, the lowest input ratio was for test #20 at 2%, and the highest was for test #24 at 45.7%. According to the detection limits stated in the manufacturers’ product information, the RATs could detect SARS-CoV-2 at 35–800 TCID_50_/mL or target virus protein at 10–25 pg/mL (Table 2). The results are assessed 5–30 min after adding the analyte (Table 2).

### 3.2. Sensitivity of RATs for SARS-CoV-2 Delta Variant Detection

To compare the sensitivity of the 27 RATs, a delta variant (lineage B.1.617.2) of SARS-CoV-2 was diluted to the indicated PFU and then examined by RT-qPCR to determine the Cq value of each sample. The Cq values were 17.1, 20.9, 24.5, 27.6, and 31.0 at 75,000, 7500, 750, 75, and 7.5 PFU (Table 3). Test #22 detected 75 PFU of delta variant in one out of the two tests but failed to detect 7.5 PFU of virus (Table 3). Tests #1, #8, #9, and #17 detected 750 PFU of delta variant in both two tests, whereas tests #7, #20, and #27 detected 750 PFU of delta variant in one out of the two tests. Tests #2, #4, #1, and #14 detected 75,000 PFU of delta variant in both two tests but failed to detect 7500 PFU. The other RATs detected 7500 PFU of delta variant. Taken together with the RT-qPCR data, our findings show that the sensitivity for delta variants of tests #1, #7, #8, #9, #17, #20, #22, and #27 is relatively high but lower than that of RT-qPCR.

## 4. Discussion

Here, we evaluated the sensitivity for the SARS-CoV-2 delta variant of 27 RATs available in Japan in September 2021. Eight RATs were able to detect at least around 750 PFU of virus (#1, #7, #8, #9, #17, #20, #22, and #27) and showed superior sensitivity to the delta variant. The detection limit, according to the manufacturers’ product information, is approximately 100 TCID_50_/mL, suggesting that the sensitivity of RATs tends to be low against the delta variant. It is important to note that we did not take into account any substances in the clinical specimens when we evaluated these RATs. Some biological components derived from human or indigenous microflora might interfere with the detection of virus antigens or cause a false-positive reaction, resulting in reduced sensitivity or specificity. We did not evaluate the false-positive rates and actual sensitivity of the RATs using clinical specimens and, therefore, careful consideration is needed when using these tests in clinical settings. Furthermore, there may be differences in sensitivity between lots and since we only tested one lot, it will be important to compare the sensitivity of different lots and confirm that the sensitivity is consistent.

We previously compared the sensitivity of RATs for SARS-CoV-2 possessing aspartic acid or glycine at position 614 of the S protein (S-614D or S-614G) [11]. In the present study, we included four of the RATs (#1, #2, #5, and #13) from that previous research, although the name of one of them was changed because of a supplier change (#5). The sensitivity of these four RATs to the delta variant was reduced to approximately one-tenth of that to the S-614D or -614G virus. These four RATs utilize monoclonal antibodies against the N protein of SARS-CoV-2, and the delta variant used in this study possessed the substitutions D63G, R203M, G215C, and D377Y in the N protein compared with the S-614D virus. These four amino acid substitutions might affect the sensitivity of the four RATs. Alternatively, the reduced sensitivity might be caused by a reduction in the particle/PFU ratio of the delta variant [20] because delta variants harboring L452R, E484Q, and P681R in the S protein efficiently infect cells due to the high affinity of the S protein for ACE2 and their high fusion efficiency [21,22]. This means that test specimens prepared at a certain PFU might contain fewer virus particles of the delta variant than of the S-614D virus. Since we adjusted the test specimens based on the virus PFU titers, the test specimens would contain less N protein, causing the reduction in sensitivity. This idea is supported by our quantification of the viral RNA by RT-qPCR: the Cq values at 7500, 750, and 75 PFU of S-614D or -614G virus were 16.9–18.0, 20.4–21.7, and 24.0–25.4, respectively, whereas those at 7500, 750, and 75 PFU of the delta variant were 20.9, 24.5, and 27.6, respectively (see Table 3 and [11]). These results show that the viral RNA-to-PFU ratio of the delta variant was approximately one-tenth smaller than that of the S-614D or -614G virus. Therefore, the apparent reduction in sensitivity of RATs is likely caused by the enhanced infectivity of the delta variant.

In this study, our evaluation of the sensitivity of RATs confirms previous findings by us [11] and others [12,13,14,15,16] that RATs give negative results for test samples when the amount of virus in the samples is low. However, RATs with superior sensitivity are useful for rapid diagnosis especially in limited resource settings where RT-qPCR is hard to perform, because they allow for immediate isolation of individuals shedding a large amount of virus. The sensitivity of influenza RATs has improved over time since their inception [23,24,25,26]; therefore, we anticipate that COVID-19 RATs that are more sensitive will be developed in the near future to support the control of COVID-19 and to help initiate treatment early after onset.

## Figures and Tables

**Table 1:** Characteristics of the rapid antigen tests for COVID-19 evaluated in this study.

No	Rapid Antigen Test	Manufacturer	Country of Origin	Clinical Use in Japan	Format ^a^	Recommended Test Sample ^b^
1	ESPLINE SARS-CoV-2	Fujirebio	Japan	Yes	Well	NP or N swab
2	ImmunoAce SARS-CoV-2	TAUNS Laboratories	Japan	Yes	Well	NP or N swab
3	Panbio^TM^ COVID-19 Ag Rapid Test Device	Abbott Diagnostics Medical	USA	Yes	Well	N swab
4	PRORAST SARS-CoV-2 Ag	ADTEC/LSI Medience	Japan	Yes	Well	NP or N swab
5	SARS-CoV-2 Rapid Antigen Test	Roche Diagnostics	Switzerland	Yes	Well	NP or N swab
6	Fuji Dry-Chem IMMUNO AG Handy COVID-19 Ag	Fujifilm	Japan	Yes	Well	NP or N swab
7	ALSONIC COVID-19 Ag	Alfresa Pharma	Japan	Yes	Well	NP or N swab
8	COVID-19 and Influenza A+B Antigen Combo Rapid Test	Nichirei Bioscience/Hangzhou AllTest Biotech	Japan/China	Yes	Well	NP or N swab
9	ImmunoArrow SARS-CoV-2	Toyobo	Japan	Yes	Well	NP or N swab
10	Check MR-COV19	Rohto Pharmaceutical	Japan	Yes	Well	NP or N swab
11	RapidTesta SARS-CoV-2	Sekisui Medical	Japan	Yes	Well	NP or N swab
12	QuickNavi-Flu+COVID19 Ag	Denka	Japan	Yes	Well	NP or N swab
13	QuickNavi -COVID19 Ag	Denka	Japan	Yes	Well	NP or N swab
14	KBM LineCheck nCoV	Kohjin Bio	Japan	Yes	Test strip	NP swab
15	BD Veritor System for Rapid Detection of SARS-CoV-2	Becton Dickinson	USA	Yes	Well + Analyzer	N swab
16	Sofia SARS Antigen FIA	Quidel	USA	Yes	Well + Analyzer	NP or N swab
17	Fuji Dri-chem immuno AG cartridge COVID-19 Ag	Fujifilm/Mizuho Medy	Japan	Yes	Well + Analyzer	NP or N swab
18	COVID-19 NP rapid test kit	Shanghai Cagenbio Science	China	No	Well	Saliva or P or O swab
19	SARS-CoV-2 Antigen Rapid Test	Zhuhai Encode Medical Engineering	China	No	Well	Saliva
20	2019-nCoV Ag rapid detection kit	Guangdong Longsee Biomedical	China	No	Well	Saliva or O or NP swab
21	Novel Coronavirus (SARS-CoV-2) Antigen Rapid Test Kit	Beijing Jinwofu Bioengineering Technology	China	No	Well	Saliva or O or NP swab
22	Saliva SARS-CoV-2(2019-nCoV) Antigen Test Kit	Jiaxing Wisetest Bio-tech	China	No	Pen	Saliva
23	Corona Virus (COVID-19) Antigen Rapid Test	Hoyotek Biomedical	China	No	Well	Saliva
24	SARS-CoV-2 Antigen Rapid Test Kit	JOYSBIO (Tianjin) Biotechnology	China	No	Well	Saliva
25	Novel coronavirus (2019-nCoV) antigen testing kit	Nanjing Norman Biological Technology	China	No	Well	Saliva
26	COVID19 antigen rapid test device	Toa Industry	Japan	No	Test strip	Saliva
27	Rabliss SARS-CoV-2 antigen detection kit COVID19 AG	Undisclosed	China	No	Well	Saliva

^a^ RATs were divided into three types based on their format: (i) well format, in which the lysed sample is dropped into the well and the reaction occurs inside a covered plastic body; (ii) test strip format, in which a test strip is soaked in lysis buffer containing the specimen or dipped in the specimen and then soaked in the lysis buffer, and the reaction occurs on the strip; or (iii) pen format, in which a test strip is dipped into the specimen and the reaction occurs on the strip. “+ Analyzer” means that these RATs need an analyzer to evaluate the result. ^b^ NP, nasopharyngeal; N, nasal; P, pharyngeal; O, oropharyngeal.

**Table 2:** Rapid antigen tests for COVID-19.

No.	Rapid Antigen Test	Input Rate (%) ^a^	Detection Limit ^b^	Time to Result (min) ^c^
1	ESPLINE SARS-CoV-2	8.0	25 pg/mL	10–30
2	ImmunoAce SARS-CoV-2	13.3	35.6 TCID_50_/test	15
3	Panbio^TM^ COVID-19 Ag Rapid Test Device	14.3	157.7 TCID_50_/mL	15–20
4	PRORAST SARS-CoV-2 Ag	18.2	42 Pfu/mL	15
5	SARS-CoV-2 Rapid Antigen Test	14.3	490 TCID_50_/mL	15–30
6	Fuji Dry-Chem IMMUNO AG Handy COVID-19 Ag	6.0	110 TCID_50_/mL	10
7	ALSONIC COVID-19 Ag	10.9	800 TCID_50_/mL	5
8	COVID-19 and Influenza A+B Antigen Combo Rapid Test	28.6	100 pg/mL	15
9	ImmunoArrow SARS-CoV-2	22.2	25 pg/mL	15
10	Check MR-COV19	21.9	100 TCID_50_/mL	15
11	RapidTesta SARS-CoV-2	21.8	110 TCID_50_/mL	10
12	QuickNavi-Flu+COVID19 Ag	12.5	53 TCID_50_/mL	10
13	QuickNavi -COVID19 Ag	12.5	53 TCID_50_/mL	10
14	KBM LineCheck nCoV	100	625 TCID_50_/mL	10
15	BD Veritor System for Rapid Detection of SARS-CoV-2	26.7	140 TCID_50_/mL	15
16	Sofia SARS Antigen FIA	34.3	113 TCID_50_/mL	15
17	Fuji Dri-chem immuno AG cartridge COVID-19 Ag	23.1	10 pg/mL	15
18	COVID-19 NP rapid test kit	22.2	N.A. ^d^	15
19	SARS-CoV-2 Antigen Rapid Test	8.6	N.A.	20
20	2019-nCoV Ag rapid detection kit	2.0	N.A.	15
21	Novel Coronavirus (SARS-CoV-2) Antigen Rapid Test Kit	11.1	100 TCID_50_/mL	15
22	Saliva SARS-CoV-2(2019-nCoV) Antigen Test Kit	100	N.A.	15
23	Corona Virus (COVID-19) Antigen Rapid Test	25	N.A.	15
24	SARS-COV-2 Antigen Rapid Test Kit	45.7	160 TCID_50_/mL	15–20
25	Novel coronavirus (2019-nCoV) antigen testing kit	22.9	121 TCID_50_/mL	15–20
26	COVID19 antigen rapid test device	100	N.A.	15
27	Rabliss SARS-CoV-2 antigen detection kit COVID19 AG	10.9	N.A.	8

^a^ For all tested RATs, 50 μL of test sample was used per test. The samples were mixed with lysis buffer (A). All or part of the lysed sample (B) was subjected to the assay. Input ratios were calculated by using the formula: volume B/(50 μL + volume A) × 100. ^b^ Detection limit (TCID_50_ or Pfu for virus titer; pg for antigen protein) is based on the information provided by the individual manufacturer. ^c^ The time required to obtain the results is based on the individual manufacturer’s instructions. ^d^ Not available.

**Table 3:** Sensitivity of rapid antigen tests for the delta variant.

No.	Rapid Antigen Test	Virus Titer Tested (PFU/Test)				
		75,000	7500	750	75	7.5
-	RT-qPCR	17.1 ^a^	20.9	24.5	27.6	31.0
1	ESPLINE SARS-CoV-2	+ ^b^	+	+	–	n.d.
2	ImmunoAce SARS-CoV-2	+	–	–	n.d.	n.d.
3	Panbio^TM^ COVID-19 Ag Rapid Test Device	+	+	–	n.d.	n.d.
4	PRORAST SARS-CoV-2 Ag	+	–	–	n.d.	n.d.
5	SARS-CoV-2 Rapid Antigen Test	n.d. ^c^	+	–	–	n.d.
6	Fuji Dry-Chem IMMUNO AG Handy COVID-19 Ag	n.d.	+	–	–	n.d.
7	ALSONIC COVID-19 Ag	n.d.	+	±	–	n.d.
8	COVID-19 and Influenza A+B Antigen Combo Rapid Test	n.d.	+	+	–	n.d.
9	ImmunoArrow SARS-CoV-2	n.d.	+	+	–	n.d.
10	Check MR-COV19	+	–	–	n.d.	n.d.
11	RapidTesta SARS-CoV-2	+	+	–	n.d.	n.d.
12	QuickNavi-Flu+COVID19 Ag	+	+	–	n.d.	n.d.
13	QuickNavi -COVID19 Ag	+	+	–	–	n.d.
14	KBM LineCheck nCoV	+	–	–	–	n.d.
15	BD Veritor System for Rapid Detection of SARS-CoV-2	+	+	–	–	n.d.
16	Sofia SARS Antigen FIA	+	+	–	–	n.d.
17	Fuji Dri-chem immuno AG cartridge COVID-19 Ag	n.d.	+	+	–	n.d.
18	COVID-19 NP rapid test kit	+	+	–	–	n.d.
19	SARS-CoV-2 Antigen Rapid Test	+	+	–	–	n.d.
20	2019-nCoV Ag rapid detection kit	+	+	±	–	n.d.
21	Novel Coronavirus (SARS-CoV-2) Antigen Rapid Test Kit	+	+	–	–	n.d.
22	Saliva SARS-CoV-2(2019-nCoV) Antigen Test Kit	n.d.	+	+	±	–
23	Corona Virus (COVID-19) Antigen Rapid Test	+	±	–	–	n.d.
24	SARS-CoV-2 Antigen Rapid Test Kit	+	+	–	–	n.d.
25	Novel coronavirus (2019-nCoV) antigen testing kit	+	+	–	–	n.d.
26	COVID19 antigen rapid test device	+	+	–	–	n.d.
27	Rabliss SARS-CoV-2 antigen detection kit COVID19 AG	n.d.	+	±	–	–

SARS-CoV-2 delta variant was examined with each RAT according to the manufacturers’ instructions. ^a^ Average Cq value of RT-qPCR (*n* = 3). ^b^ Two independent experiments were performed: ‘+’ indicates both were positive, ‘±’ indicates one was positive and the other was negative, ‘–’ indicates both were negative. ^c^ Not done.

## Data Availability

Not applicable.
